# Relationship between acromial morphological variation and subacromial impingement: A three-dimensional analysis

**DOI:** 10.1371/journal.pone.0176193

**Published:** 2017-04-25

**Authors:** Xinyu Li, Wei Xu, Ning Hu, Xi Liang, Wei Huang, Dianming Jiang, Hong Chen

**Affiliations:** 1 Department of Pharmacy, The First Affiliated Hospital of Chongqing Medical University, Chongqing, China; 2 Department of Orthopedics, The First Affiliated Hospital of Chongqing Medical University, Chongqing, China; Harvard Medical School/BIDMC, UNITED STATES

## Abstract

Purpose: To evaluate the association of acromial morphology and subacromial impingement. Methods: Bilateral shoulder computed tomography was performed in 138 patients who received shoulder arthroscopy. Measured parameters included: acromial tilt (AT), modified acromial tilt (mAT), acromial slope (AS), acromiohumeral interval (AHI), lateral acromial angle (LAA), acromial index (AI), critical shoulder angle (CSA), acromial anterior protrusion (AAP), and acromial inferior protrusion (AIP). Acromial morphological characteristics were compared between groups. Side-to-side differences were assessed between affected and non-affected shoulders. Intra- and inter-observer agreements for each parameter were calculated. Results: AT (25.90 *vs*. 29.41°), mAT (18.88 *vs*. 22.64°), and AHI (5.46 *vs*. 6.47 mm) were significantly smaller in impinged patients. The impingement group demonstrated significantly larger AI (63.50 *vs*. 59.84%), CSA (31.78 *vs*. 28.74°), AAP (7.13 *vs*. 5.32 mm), and AIP (5.51 *vs*. 4.04 mm). Regarding side-to-side comparison, the acromial morphology was significantly different between the affected and non-affected shoulders in impinged patients, while the difference was slight and insignificant in control patients. All, except AS and LAA, measured parameters demonstrated good intra- and inter-observer agreements. Conclusions: Three-dimensional reconstructed CT scan is a reliable method to measure shoulder morphology. The acromial morphological variation is related with sub acromial impingement, however, the causal relationship of them should be further explored.

## Introduction

Shoulder pain is the second-most common complaint faced by orthopedists. Subacromial impingement and related rotator cuff disease is reported to be the leading cause [1]. The pathogenesis of subacromial impingement is multifactorial, possible theories ranging from intrinsic tendon degeneration to extrinsic tendon compression caused by anatomical and/or kinematic factors [2]. Among the pathological factors, abnormal acromial morphology and resultant subacromial space narrowing has long been considered an important cause of subacromial impingement. Correspondingly, acromioplasty, which consists of bone removal from the anterior and lateral margins of the acromion and release of the coracoacromial (CA) ligament, has become a popular surgical strategy for those patients [3]. However, conflicting results exist regarding this procedure, and some researches suggested against the theory of extrinsic rotator cuff compression and against the necessity of acromioplasty [4,5].

Numerous studies have been conducted to explore the role of acromial morphological variation in the pathogenesis of subacromial impingement [6–10]. However, their results were inconsistent and reliabilities were not satisfactory due to the difficulty in radiographic measurement [11]. In this study, we have employed three-dimensional reconstructed computed tomography (CT) technique to evaluate the association between acromial morphology and subacromial impingement. In addition to commonly used patient-control comparison strategy, a direct side-to-side difference between the affected and non-affected shoulders was also calculated to explore the causal relationship of these two entities.

## Methods

The Institutional Review Board of the first affiliated hospital of Chongqing medical university approved the study protocol S1 and S2 Files. Written informed consent was obtained from all individual participants included in the study. Patients who received shoulder arthroscopy at one institution between July 2014 and June 2016 were enrolled. Data were collected prospectively and reviewed retrospectively. Authors of the present study didn’t have access to information that could identify individual participants during or after data collection.

Patients who had signs and symptoms consistent with the clinical diagnosis of unilateral subacromial impingement with or without RCT were enrolled in impingement group. Those diagnosed as calcifying tendinitis, frozen shoulder, or recurrent anterior dislocation with intact rotator cuff were enrolled in control group. Exclusion criteria included: traumatic rotator cuff tear, glenohumeral osteoarthritis, and previous shoulder surgery history.

### Three-dimensional CT scan

**Fig 1:**
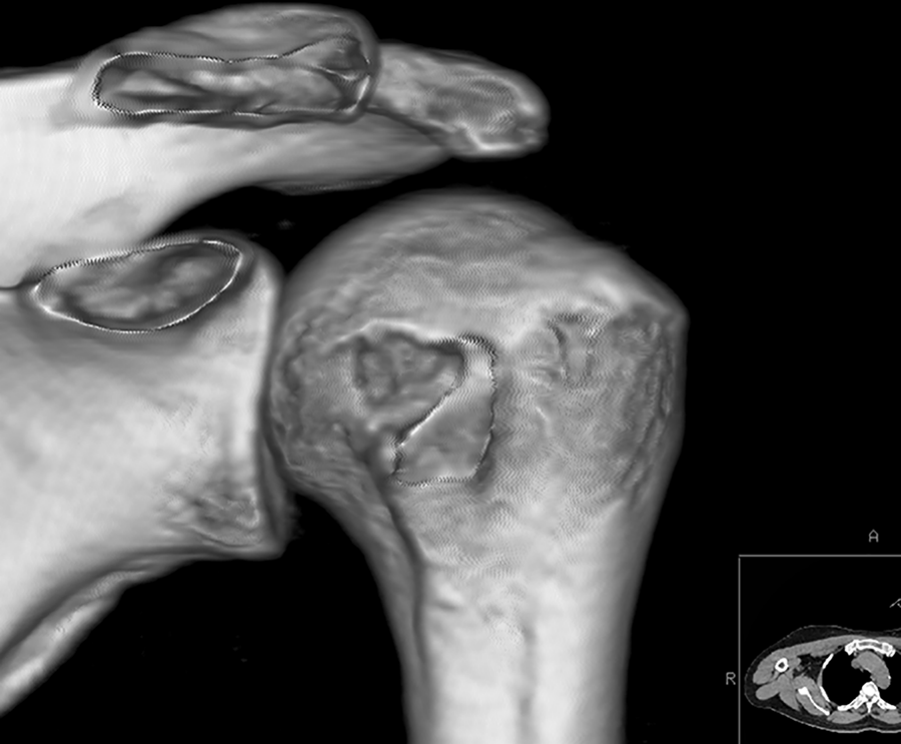
True anteroposterior view. The plane of the scapula was derotated so that the anterior and posterior glenoid edges were overlapped.

**Fig 2:**
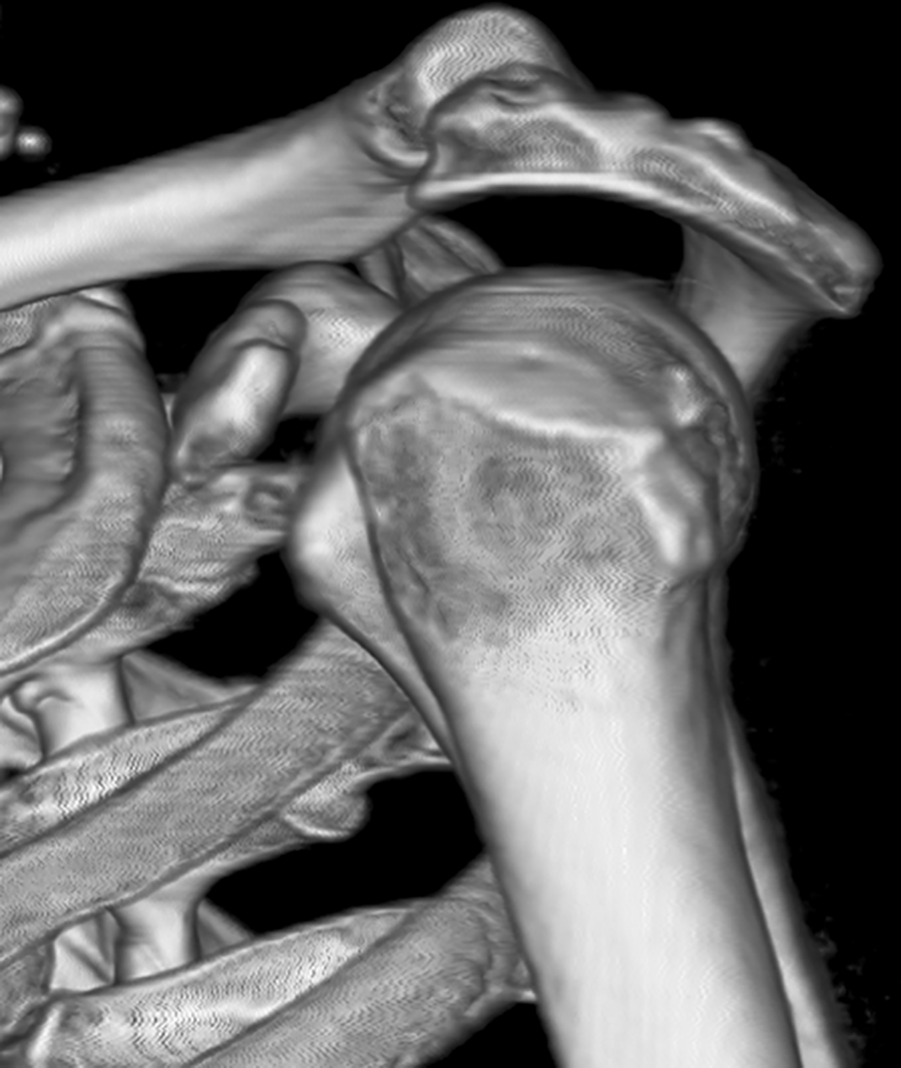
Standard outlet view. The view perpendicular to the glenoid plane with overlapped medial and lateral scapular margins.

**Fig 3:**
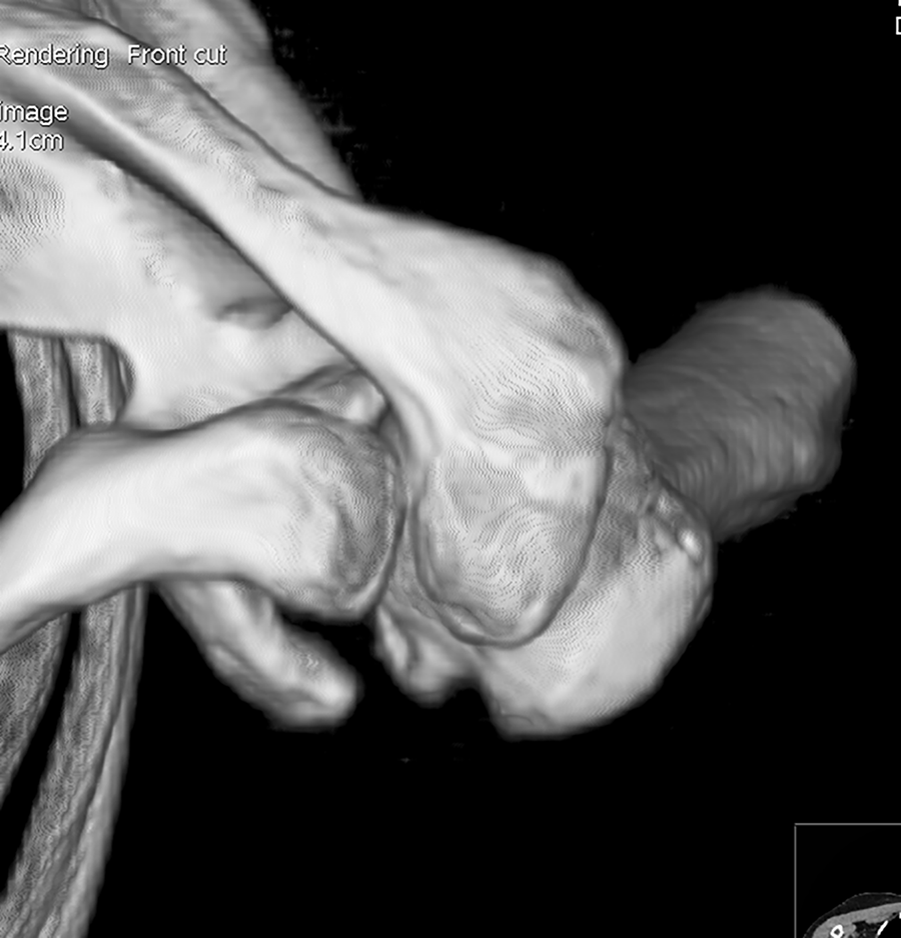
Superior view. The viewing angle was downwardly perpendicular to the plane of the acromioclavicular joint.

**Fig 4:**
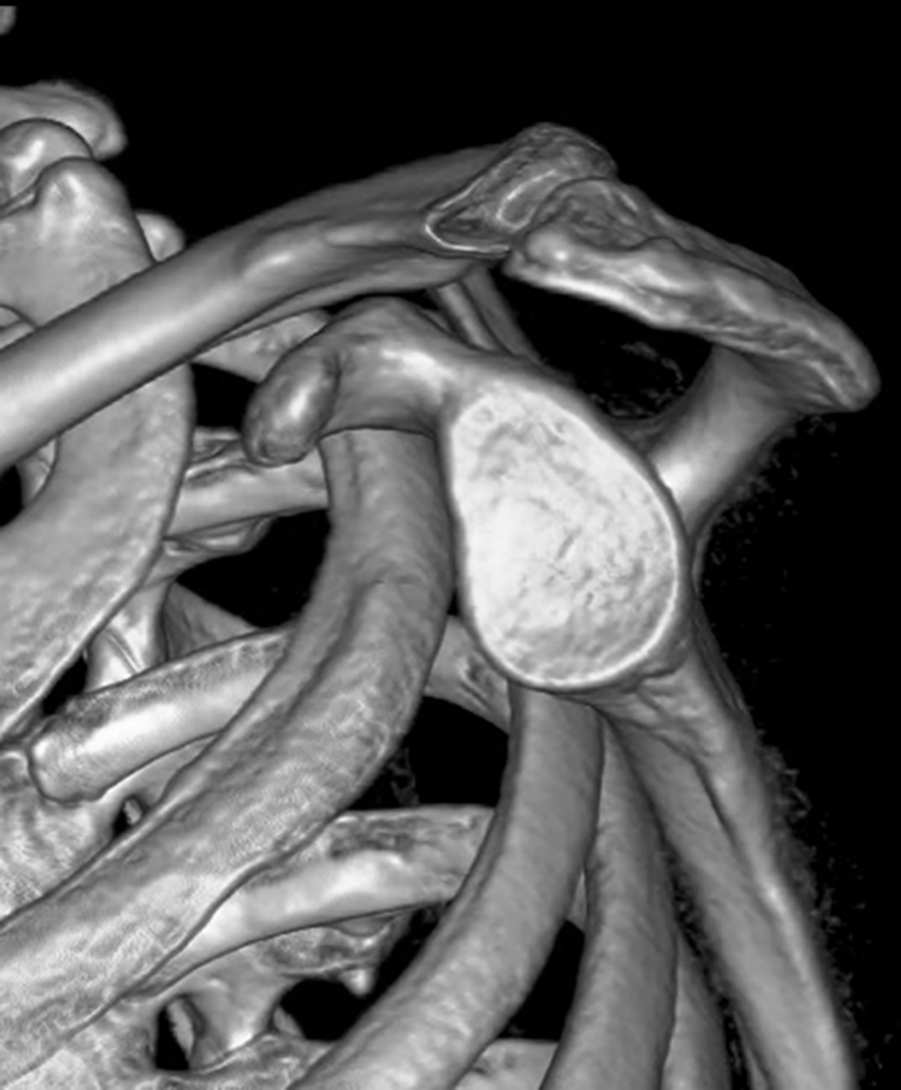
Humeral head removal. The humeral head was removed to facilitate measurement.

All participants underwent bilateral shoulder CT scans (LightSpeed VCT, GE Healthcare, London, UK) before arthroscopic surgery. Digital Imaging and Communications in Medicine (DICOM) data were used to acquire multi-planar reformatted three-dimensional images (slice thickness, 1.25mm). GE Advantage Workstation for diagnostic imaging software (GE AW 4.6) was employed to perform image processing and measurement. By rotating and tilting these reformatted images, it was possible to measure different parameters in true anteroposterior (AP) view, standard outlet view, and superior view. On the true AP view, the plane of the scapula was derotated so that the anterior and posterior glenoid edges were overlapped (Fig 1). The standard outlet view was defined as the view perpendicular to the glenoid plane with overlapped medial and lateral scapular margins (Fig 2). In the superior view, the viewing angle was downwardly perpendicular to the plane of the acromioclavicular joint (Fig 3). The removal of humeral head or image cut from different directions were performed when needed (Fig 4).

### Measurements

Anatomic landmarks were marked manually using the GE AW 4.6 workstation software by two independent readers. Subsequently, the following angles and distances were measured with the same software. Each reader measured twice the same parameter, the average value was used for further calculation.

**Fig 5:**
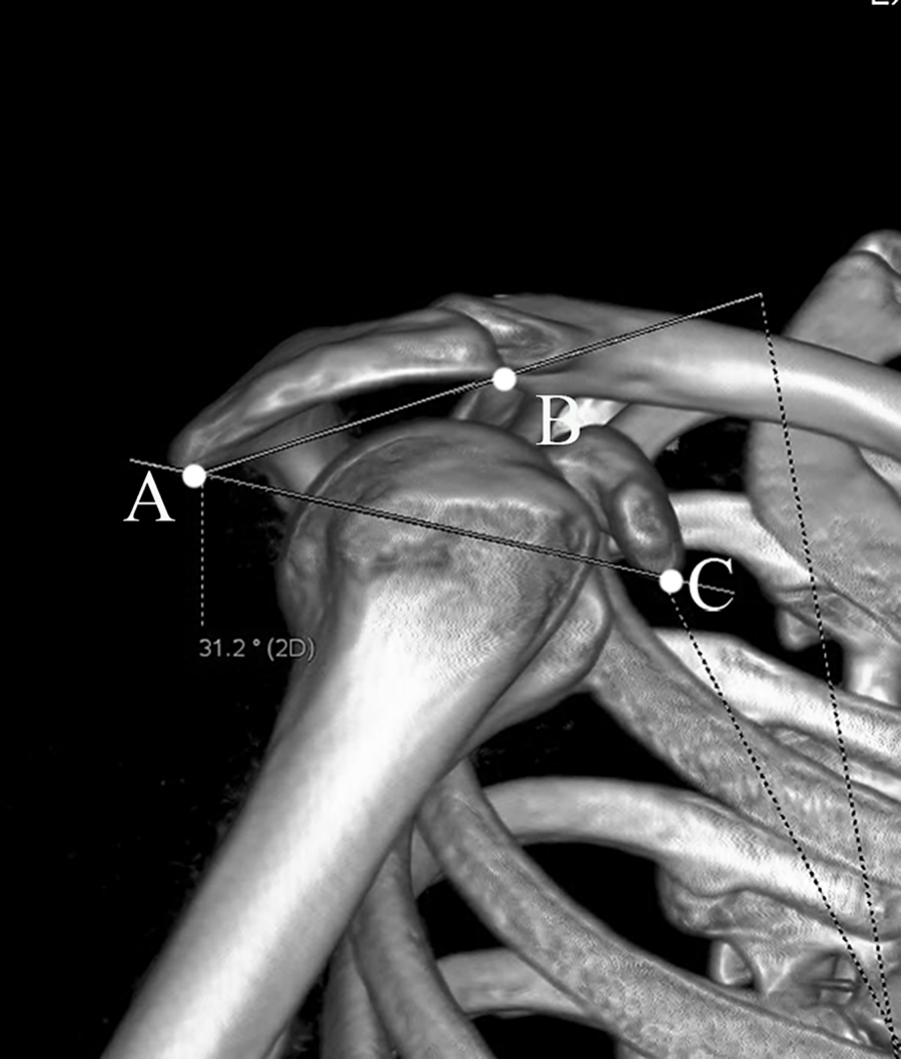
The measurement of acromial tilt (AT) in standard outlet view. The postero-inferior edge (point A), antero-inferior edge (point B), and the inferior tip of the coracoid process (point C) were marked. The angle ∠BAC represented the AT angle.

**Fig 6:**
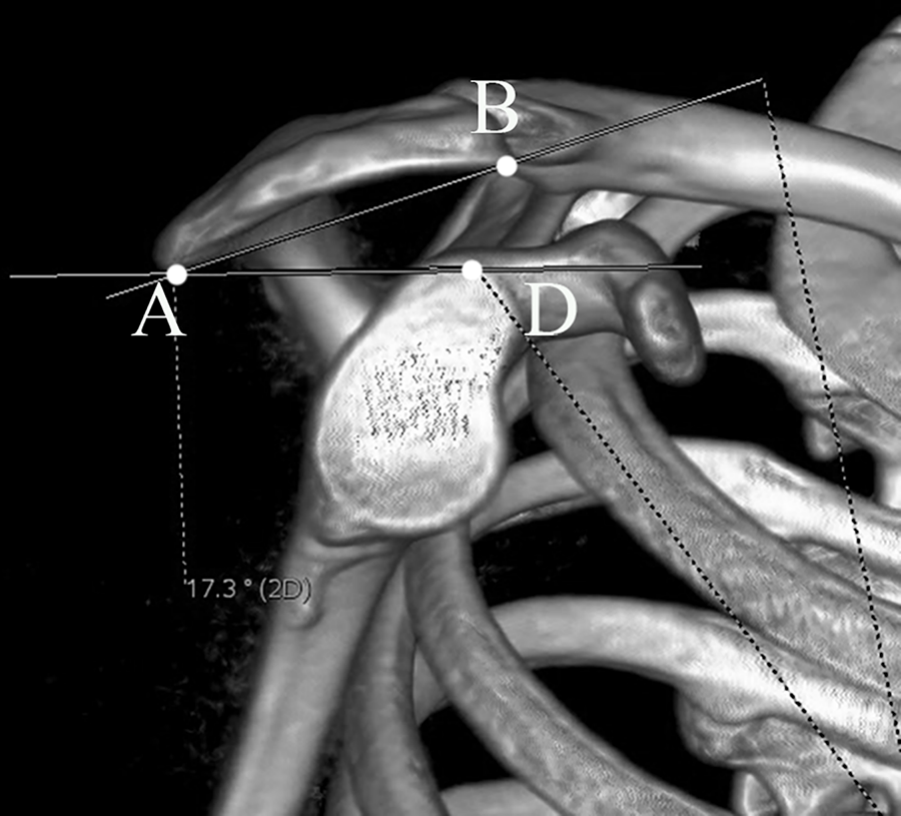
The measurement of modified acromial tilt (mAT) in standard outlet view. The supraglenoid tubercle was marked as point D, the angle ∠BAD defined mAT.

The acromial tilt (AT) was measured on outlet view image [12,13]. The postero-inferior acromial edge (point A), antero-inferior acromial edge (point B), and the inferior tip of the coracoid process (point C) were marked. Then points A-B and A-C were connected. The angle ∠BAC represented the AT angle (Fig 5). Because the AT value is influenced by the shape and size of the coracoid process, we have introduced modified acromial tilt (mAT) for the first time to avoid potential bias. When measuring mAT, the inferior tip of the coracoid process (point C) was replaced by the supraglenoid tubercle (point D). Thus, the angle ∠BAD defined mAT (Fig 6).

**Fig 7:**
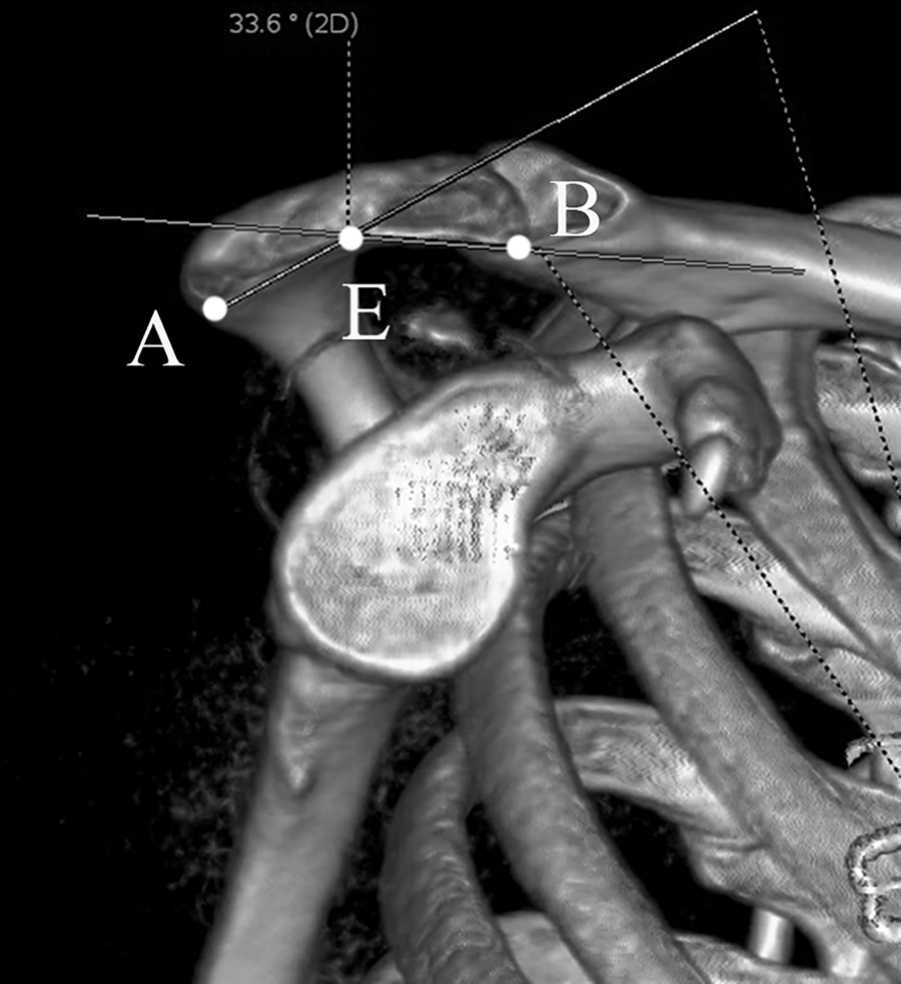
Acromial slope (AS) measurement in standard outlet view. Points A, B and the midway point on the inferior aspect of acromion (point E) were marked. The supplementary angle of ∠BEA represented the AS.

**Fig 8:**
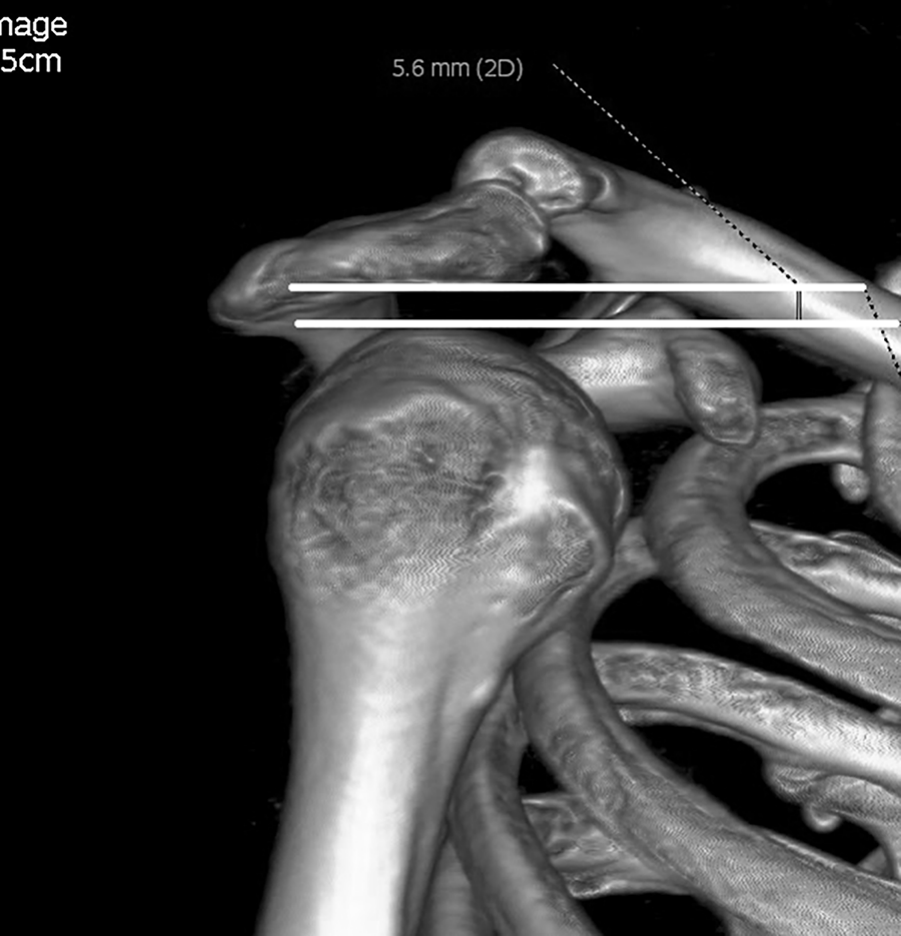
Acromiohumeral interval (AHI) measurement in standard outlet view. The distance between the inferior aspect of the acromion and the most superior point of the humeral head was AHI.

The acromial slope (AS) was also measured on outlet view image [12,13]. Points A, B and the midway point on the inferior aspect of acromion (point E) were marked. The supplementary angle of ∠BEA represented the AS (Fig 7). On outlet view image, the acromiohumeral interval (AHI) was the distance between the inferior aspect of the acromion and the most superior point of the humeral head [14] (Fig 8).

**Fig 9:**
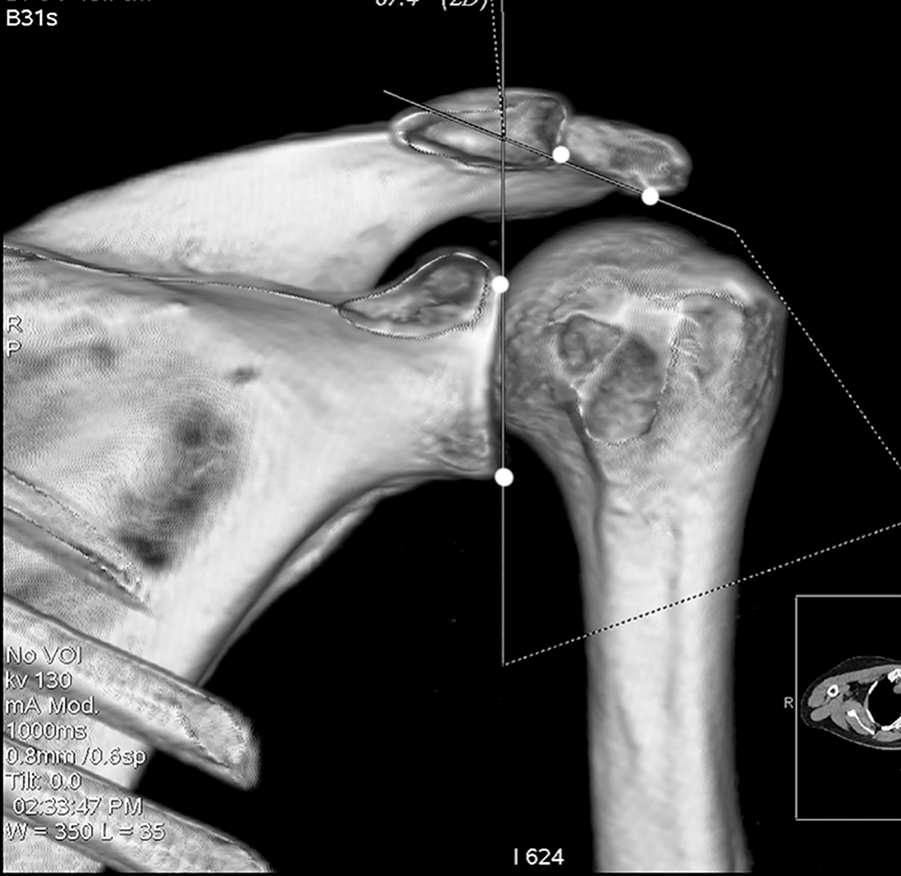
Lateral acromial angle (LAA). One line was drawn along the superior-most and inferior-most points of the glenoid fossa, another line was drawn parallel to the acromion undersurface. The angle formed by these 2 lines represented the LAA.

**Fig 10:**
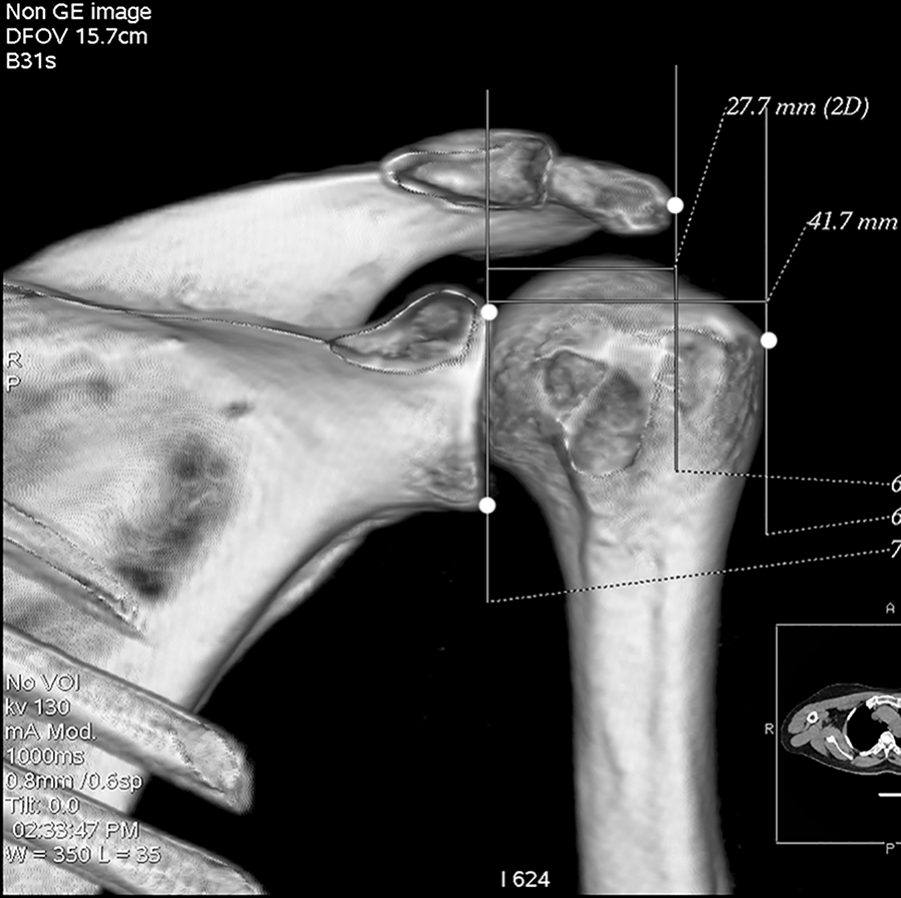
Acromion index (AI). The AI was defined as the ratio of the distance from the glenoid plane to the lateral acromion to the distance from the glenoid plane to the lateral aspect of the humeral greater tubercle.

**Fig 11:**
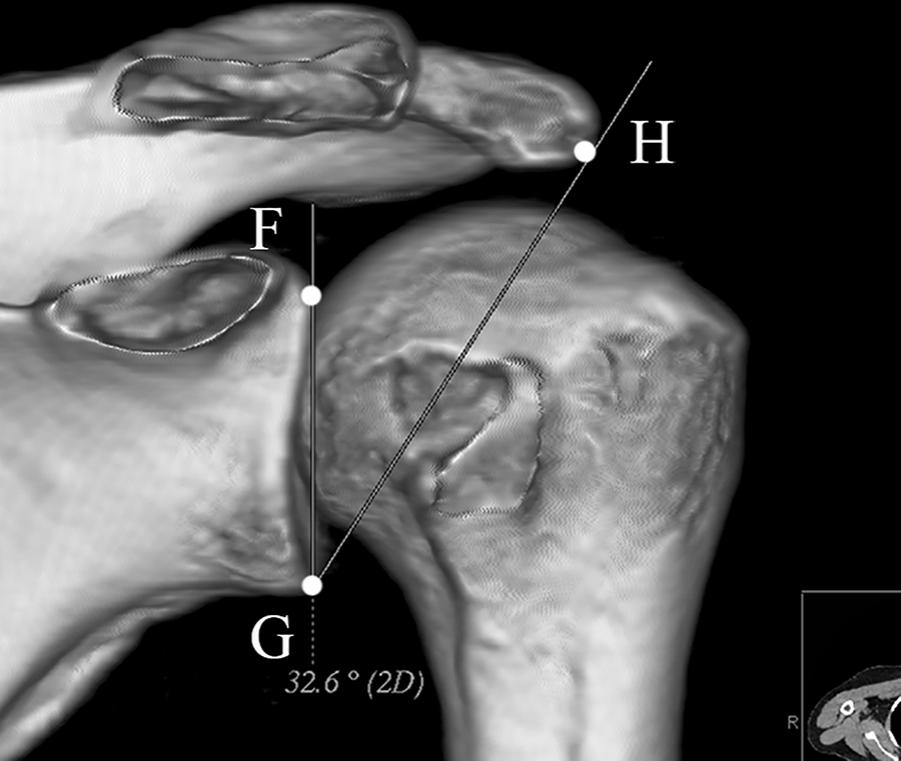
Critical shoulder angle (CSA). The superior-most (point F) and inferior-most (point G) points of the glenoid fossa, as well as the most inferolateral point of the acromion (point H) were marked. The angle ∠FGH represented the CSA.

Lateral acromial angle (LAA), acromion index (AI), and critical shoulder angle (CSA) were all measured in true AP view. When measuring the LAA, one line was drawn along the superior-most and inferior-most points of the glenoid fossa, another line was drawn parallel to the acromion undersurface. The angle formed by these 2 lines represented the LAA [15] (Fig 9). The AI was defined as the ratio of the distance from the glenoid plane to the lateral acromion to the distance from the glenoid plane to the lateral aspect of the humeral greater tubercle [16] (Fig 10). In order to measure the CSA, the superior-most (point F) and inferior-most (point G) points of the glenoid fossa, as well as the most inferolateral point of the acromion (point H) were marked. The angle ∠FGH represented the CSA [17] (Fig 11).

**Fig 12:**
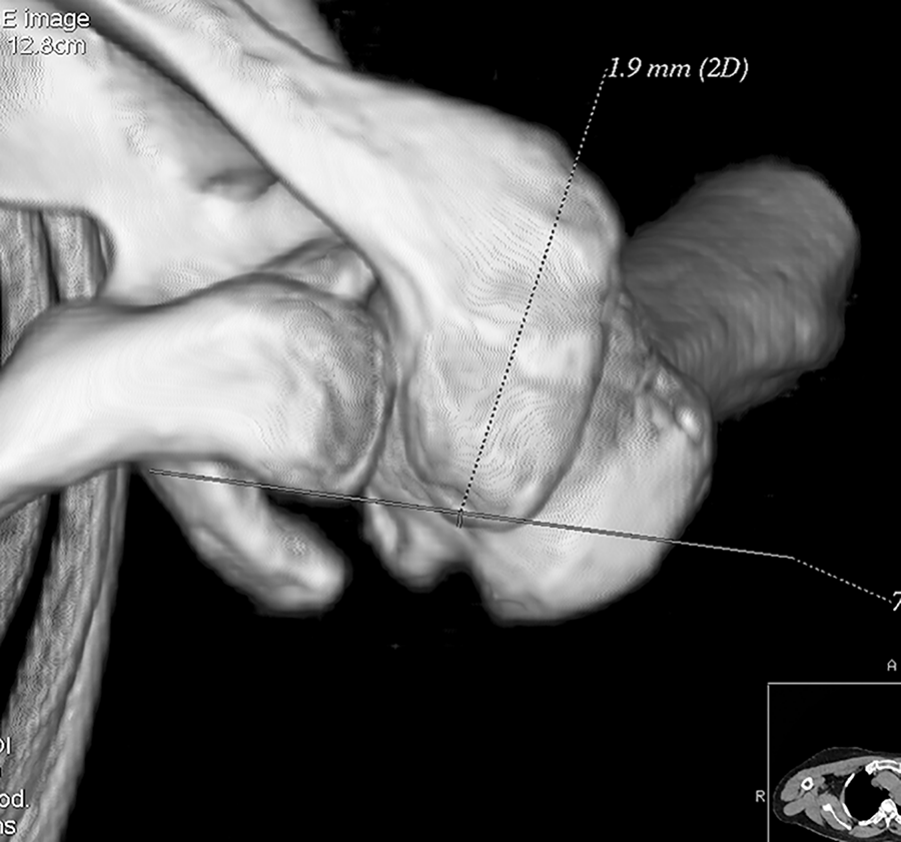
Acromial anterior protrusion (AAP). AAP was measured on the superior view. A line coincident with the anterior aspect of the distal clavicle was drawn. The distance from the most anterior point of the acromion to this line was the AAP.

**Fig 13:**
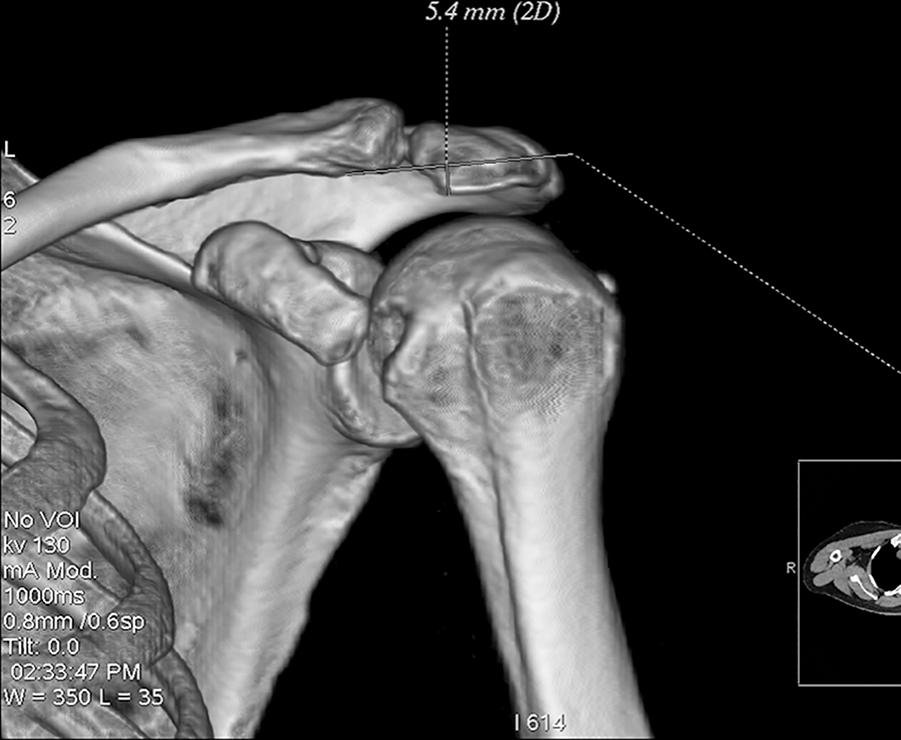
Acromial inferior protrusion (AIP). AIP was measured on the true anteroposterior view. It was defined as the distance from the most inferior point of the anterior acromion to the line which was coincident with the inferior aspect of the distal clavicle.

The acromial anterior protrusion (AAP) and acromial inferior protrusion (AIP) were also introduced for the first time to directly observe the acromial protrusions. AAP was measured on the superior view. A line coincident with the anterior aspect of the distal clavicle was drawn. The distance from the most anterior point of the acromion to this line was AAP (Fig 12). AIP was measured on the true AP view. It was defined as the distance from the most inferior point of the anterior acromion to the line which was coincident with the inferior aspect of the distal clavicle (Fig 13).

### Statistics

Statistical evaluation was performed with SPSS software version 17.0 (SPSS Inc, Chicago, IL). Descriptive statistics (means and standard deviations) were obtained for quantitative variables. Two-tailed paired and unpaired samples student *t* tests were employed for side to side comparison and comparison of impingement and control affected acromial morphologies, respectively. The significance level was set at *P*<0.05. To determine whether acromial morphology differed in impinged and non-impinged patients, comparisons have been performed between the affected shoulders of the impingement and control groups. Besides, the side-to-side comparison has been performed in all patients to explore the causal relationship of acromial morphology and subacromial impingement. To evaluate the intra- and inter-observer agreement of the measurements, the intra-class correlation coefficients (ICCs) of each parameter were calculated using the two-way mixed effects model. We considered ICCs of 0.7 or higher to be sufficient for the reliability.

## Results

### Patient demographics

Between September 2014 and August 2016, a total of 138 consecutive patients underwent shoulder arthroscopy in our institution. Among them, 62 patients were diagnosed as unilateral subacromial impingement with or without RCT (29 males and 33 females, average age, 51 years, range 44–71 years) and underwent arthroscopic decompression as well as rotator cuff repair when necessary (impingement group); the remaining 76 patients (34 males and 42 females, average age, 47 years, range 16–73 years) were diagnosed as calcifying tendinitis, frozen shoulder, or recurrent anterior dislocation and underwent relevant arthroscopic surgeries (control group).

In the impingement group, the dominant shoulder was mostly affected (87%), and the average duration of symptom was 6 months (range, 3–8 months). In the control group, also the dominant side (67%) was more frequently involved than the non-dominant side, and the average symptom duration was 11 months (range, 7–18 months).

### Acromial morphological differences between impingement and control patients

The mean acromial tilt angle (AT) was significantly smaller in the impingement patients than in control patients (25.90 ± 4.27 *vs*. 29.41 ± 4.07°, *P* = 0.0026). Also, the average modified AT angle (mAT) was significantly smaller in impingement patients (18.88 ± 3.96 *vs*. 22.64 ± 3.78°, *P* = 0.0006). However, no significant difference of acromial slope (AS) existed between groups (24.20 ± 8.47 *vs*. 25.40 ± 5.80°, *P* = 0.1054). The average acromiohumeral interval (AHI) in impingement patients was 5.46 ± 1.37 mm, which was significantly smaller than in control patients (6.47 ± 1.90 mm, *P* = 0.0473).

There was 3° difference of lateral acromial angle (LAA) between the two groups, however, this difference didn’t reach significance (75.24 ± 8.53 *vs*. 78.48 ± 7.83°, *P* = 0.1405). The acromial index (AI) was also slightly and insignificantly larger in impingement patients (63.50 ± 7.14 *vs*. 59.84 ± 9.86%, *P* = 0.1336). The impingement patients demonstrated a significantly larger critical shoulder angle (CSA) than their control counterparts (31.78 ± 4.64 *vs*. 28.74 ± 4.70°, *P* = 0.0183).

The acromial anterior protrusion (AAP) and acromial inferior protrusion (AIP) distances were significantly larger in impingement group (AAP: 7.13 ± 4.65 *vs*. 5.32 ± 3.05 mm, *P* = 0.0190; AIP: 5.51 ± 2.88 *vs*. 4.04 ± 2.20 mm, *P* = 0.0316) (Table 1).

**Table 1:** Acromial morphological variations between impingement and control patients (mean±SD).

	Impingement	Control	*P* value*
**AT (°)**	25.90 ± 4.27	29.41 ± 4.07	0.0026
**mAT (°)**	18.88 ± 3.96	22.64 ± 3.78	0.0006
**AS (°)**	24.20 ± 8.47	25.40 ± 5.80	0.6153
**AHI (mm)**	5.46 ± 1.37	6.47 ± 1.90	0.0473
**LAA (°)**	75.24 ± 8.53	78.48 ± 7.83	0.1405
**AI (%)**	63.50 ± 7.14	59.84 ± 9.86	0.1336
**CSA (°)**	31.78 ± 4.64	28.74 ± 4.70	0.0183
**AAP (mm)**	7.13 ± 4.65	5.32 ± 3.05	0.0190
**AIP (mm)**	5.51 ± 2.88	4.04 ± 2.20	0.0316

*: Unpaired samples student *t* test between affected shoulders of impingement and control patients

### Side-to-side differences of acromial morphological parameters

In the impingement group, the AT in the affected side (25.90 ± 4.27°) was significantly smaller than in the contralateral side (28.09 ± 3.76°, *P* = 0.0054). Correspondingly, there was also significant difference in mAT value (affected side: 18.73 ± 4.00°, non-affected side: 20.60 ± 3.36°, *P* = 0.0395). Compared with the normal side, the affected humeral head migrated superiorly for 1 mm, which also reached significance (AHI: 5.46 ± 1.37 *vs*. 6.43 ± 2.30 mm, *P* = 0.0263). Both AAP and AIP were significantly increased in the affected shoulder, indicating anterior and inferior protrusion of impinged acromion (AAP: 7.13 ± 4.65 *vs*. 4.40 ± 3.05 mm, *P* = 0.0125; AIP: 5.51 ± 2.88 *vs*. 4.16 ± 2.47 mm, *P* = 0.0015). No significant difference has been demonstrated for other parameters including AS, LAA, AI, and CSA (Table 2). Within the control group, although slight differences existed between the affected and non-affected side, none of these varieties reached significance (Table 2).

**Table 2:** Side-to-side differences of acromial morphological parameters (mean±SD).

	Impingement group			Control group		
	Affected side	Non-affected side	*P* value*	Affected side	Non-affected side	*P* value*
**AT (°)**	25.90±4.27	28.09±3.76	0.0054	29.41±4.07	30.04±4.94	0.4210
**mAT (°)**	18.73±4.00	20.60±3.36	0.0395	22.64±3.78	23.27±3.93	0.3576
**AS (°)**	24.20±8.47	24.82±8.28	0.6171	27.53±8.15	26.22±8.12	0.3344
**AHI (mm)**	5.46±1.37	6.43±2.30	0.0263	6.47±1.90	6.66±1.60	0.3700
**LAA (°)**	75.24±8.53	77.60±6.61	0.2333	78.48±7.83	79.01±6.46	0.6383
**AI (%)**	63.50±7.14	62.04±7.03	0.2125	58.75±8.78	57.19±9.52	0.0576
**CSA (°)**	31.78±4.64	31.11±3.87	0.4003	28.73±4.70	28.53±4.26	0.6744
**AAP (mm)**	7.13±4.65	4.40±3.05	0.0125	4.91±3.44	4.79±2.91	0.8351
**AIP (mm)**	5.51±2.88	4.16 ±2.47	0.0015	3.88±2.40	3.76±2.05	0.7338

*: Paired samples student *t* test between affected and non-affected shoulders

### Intra- and inter-observer agreement

With respect to AT, mAT, AHI, AI, and CSA, the ICCs were all above 0.90, suggesting very high intra- and inter-observer reliabilities for these parameters. Regarding AAP and AIP, the ICCs were ranging from 0.73 to 0.80. The intra- and inter-observer agreements for AS and LAA were moderate, with ICCs ranging from 0.59 to 0.69 (Table 3).

**Table 3:** Intra- and inter-observer agreement for acromial morphological parameters.

	Intra-observer agreement			Inter-observer agreement		
	1^st^ evaluation*	2^nd^ evaluation*	ICC	1^st^ observer*	2^nd^ observer*	ICC
**AT (°)**	28.92±4.27	29.02±4.76	0.96	28.81±4.07	29.13±4.94	0.95
**mAT (°)**	21.73±3.00	22.01±2.96	0.94	21.94±4.08	21.80±3.91	0.94
**AS (°)**	26.20±9.07	24.89±8.21	0.60	26.53±8.35	24.56±9.12	0.59
**AHI (mm)**	6.46±1.87	6.20±2.31	0.88	6.35±1.97	6.31±2.64	0.87
**LAA (°)**	78.94±8.53	77.90±6.21	0.69	77.98±7.33	78.86±6.16	0.67
**AI (%)**	60.10±8.14	59.04±9.03	0.91	58.99±9.00	60.15±9.12	0.89
**CSA (°)**	30.05±5.64	30.11±5.87	0.94	29.93±5.70	30.23±5.26	0.90
**AAP (mm)**	5.13±4.17	4.97±3.51	0.80	4.91±3.04	5.19±3.21	0.76
**AIP (mm)**	4.31±2.18	4.19±2.49	0.78	4.18±2.49	4.32±2.25	0.73

*: Values were presented as means ± SDs; ICC: intra-class correlation coefficient

## Discussions

Pathological factors that are considered to contribute to subacromial impingement can be categorized into extrinsic and intrinsic ones. Extrinsic factors impose direct compression on the rotator cuff tendons in the subacromial space, including alignment, anatomical, kinematic, and so on. Intrinsic factors are those that contribute to rotator cuff tendon degeneration, consisting of reduced vascularity, overload, etc [5]. Previously, abnormal acromial morphology has been believed to be the primary predisposing factor of subacromial rotator cuff compression, and addressing this extrinsic mechanism by acromioplasty was a common strategy. However, questioning with regard to the efficacy and necessity of acromioplasty has never ceased [3–5, 18–23]. This in turn makes researchers reconsider the role of anatomical factor in the development of subacromial impingement. In the present study, the morphological acromial differences between impingement and control patients indicate that the impinged acromia are more prominent anteriorly and inferiorly. These results were consistent with previous reports [12,16,17,24]. Nevertheless, these data could only support the correlation, but not the causal relationship, of acromial morphology and subacromial impingement.

Subsequently, side-to-side comparison showed that there were significant morphological differences between the affected and non-affected shoulders in impingement patients, while the differences were negligible in control patients. These findings may provide support to the intrinsic factor theory in the development of subacromial impingement: first, rotator cuff tendon degeneration leads to unbalanced force couples around the shoulder and resultant antero-superior glenohumeral instability; then, as a compensation and restriction mechanism to this instability, bone spurs gradually grow along the coracoacromial arch, creating a deformed acromion; in this case, it’s quite rational to expect a morphological difference between the affected and non-affected acromia in impingement patients. Based on this speculation, the acromial morphological variation is the consequence, rather than the cause, of the subacromial impingement [25]. Accordingly, acromioplasty is not only incapable of eliminating the impingement source, but also undermining the glenohumeral stabilizing mechanism, which may further aggravate rotator cuff injury [26]. Thus, the key in the subacromial impingement treatment is to restore the integrity and function of rotator cuff tendon, but not acromioplasty.

The majority of the morphometric parameters used in the present study have been well established and widely used in previous reports. Among them, AT and AS were index of acromial inferior protrusion, AHI indicated the superior migration of humeral head, and LAA, AI, and CSA represented the lateral prominence of acromion [12,16,17,24]. However, the agreement for those parameters measured on X-rays was poor [11]. In the current study, the employment of three-dimensional reconstructed CT scan has largely increased the reliability and reproducibility of the measurement. For example, Besides, with image processing technique, some special views and parameters could be used. To our knowledge, three novel indexes were employed for the first time to depict the acromial shape. Modified AT describes the relationship between the anterior acromion and the supraglenoid tubercle (Fig 2B). This parameter was introduced to replace AT because the supraglenoid tubercle has a more constant anatomical relationship with the acromion than the tip of the coracoid process. With ICC of 0.94, the reliability and reproducibility of mAT was well proven. Besides, AAP and AIP have been firstly employed to describe the anterior and inferior protrusions of anterior acromion compared to the distal clavicle (Fig 5A and 5B). Again, high intra- and inter-observer agreements suggested good reliabilities of these two parameters.

Several limitations exist in the study. First, the control group consisted of patients with frozen shoulder, calcifying tendinitis, and recurrent shoulder dislocation. This heterogeneity may potentially bias the morphometric results. However, no previous articles have reported associations between these pathologies and abnormal acromial morphology. Thus, those patients could be regarded as normal with respect to the the shape of acromion. Second, impingement patients with or without degenerative rotator cuff tears were not discriminated when performing analysis. From the authors’ perspective, the existence of degenerative rotator cuff tear could only partially reflect the duration and severity of impingement, but not influence the result fundamentally. Third, our study was a cross-sectional study which could only describe the acromial morphology at a specific time point. A longitudinal observational study would be ideal to confirm the causal relationship of acromial shape and subacromial impingement.

## Conclusions

Three-dimensional reconstructed CT scan is a reliable method to measure shoulder morphology. The impinged acromion is more prominent anteriorly and inferiorly than normal. The acromial morphological variation is related with subacromial impingement, however, the causal relationship of them should be further explored.

## Supporting information

S1 File IRB approval english version. Institutional review board approval (English translation version).
(PDF)

S2 File IRB approval original version. Institutional review board approval (Chinese version).
(PDF)
