# Supplementary material for: Relationship between acromial morphological variation and subacromial impingement: A three-dimensional analysis
Source: PLoS One. 2017 Apr 25;12(4):e0176193. doi: 10.1371/journal.pone.0176193 (PMC5404845; doi:10.1371/journal.pone.0176193)
Supplement: S1 File — Institutional review board approval (English translation version). (PDF) [file pone.0176193.s001.pdf]

Institutional Review Board Approval

Applicant：Chen Hong

Project name：Relationship between acromial morphological variation and subacromial impingement syndrome

Applicant date：2014.2.26

Issue date：2014.3.1

After reviewed by the institutional review board (IRB) of the first affiliated hospital of Chongqing medical university, the above project is approved to be conducted. IRB number: 20140312.

IRB of the first affiliated hospital of Chongqing medical university

2014.3.1
