# Supplementary material for: Relationship between acromial morphological variation and subacromial impingement: A three-dimensional analysis
Source: PLoS One. 2017 Apr 25;12(4):e0176193. doi: 10.1371/journal.pone.0176193 (PMC5404845; doi:10.1371/journal.pone.0176193)
Supplement: S2 File — Institutional review board approval (Chinese version). (PDF) [file pone.0176193.s002.pdf]

重庆医科大学附属第一医院伦理委员会
审批意见书

申请人：陈虹

申请审批项目名称：肩峰形态与肩峰下撞击综合征的相关性研究

审查日期：2014.2.26

批准日期：2014.3.1

本项目所提交的资料经伦理委员会审查，符合伦理要求，同意实施。

批准号：20140312。

重庆医科大学附属第一医院伦理委员会

重庆医科大学附属第一医院
伦理委员会

2014.3.1
